# Early Failure of Primary Total Knee Arthroplasty Due to Massive Osteolysis Caused by Bio-Absorbable Interference Screws

**DOI:** 10.7759/cureus.38143

**Published:** 2023-04-26

**Authors:** Mukund Reddy V Galiveeti, Khaldoun El-Abed, Riaz Ahmad

**Affiliations:** 1 Trauma and Orthopaedics, University Hospitals Bristol and Weston, NHS Foundation Trust, Weston-super-Mare, GBR

**Keywords:** anterior cruciate ligament reconstruction, total knee arthroplasty, osteolysis, interference screws, bio-absorbable

## Abstract

We report an unusual case in which an unabsorbed bio-absorbable screw in the tibial tunnel of anterior cruciate ligament reconstruction (ACLR) performed 11 years ago caused massive osteolysis and subsequent failure of total knee arthroplasty (TKA). ACLR was performed using suspensory fixation on the femoral side and a bio-absorbable interference screw on the tibial side. Fragmentation of the bio-absorbable screw at the time of tibial component implantation is thought to have evoked an accelerated inflammatory response, causing osteolysis, which finally resulted in early failure of the TKA.

## Introduction

The outcome of total knee arthroplasties (TKAs) is excellent, with a 95% survival at 15 years [1]. The short- and midterm subjective scores and functional outcomes appear to be comparable in TKA alone, and TKA with a history of anterior cruciate ligament reconstruction (ACLR). However, patients with prior ACLR may be more at risk for revision of TKA [2].

Graft fixation in ACLR is a weak link, particularly at the tibia, as the tunnel is nearly collinear with the pathway of the graft during extension [3]. An interference screw is frequently used for fixation in the tibial tunnel. Bio-absorbable screws are sometimes preferred over metallic screws, due to their ability to reduce stress shielding, reduced the need for implant removal and less distortion with MRI [4]. The main disadvantage of bio-absorbable screws is the undesirable biological response in the form of absorption-related intra-osseous tibial tunnel cysts [3,5].

We report a case in which an unabsorbed bio-absorbable screw used for ACLR 11 years ago led to massive osteolysis, loosening of the tibial component and ultimately TKA revision.

## Case presentation

**Figure 1:**
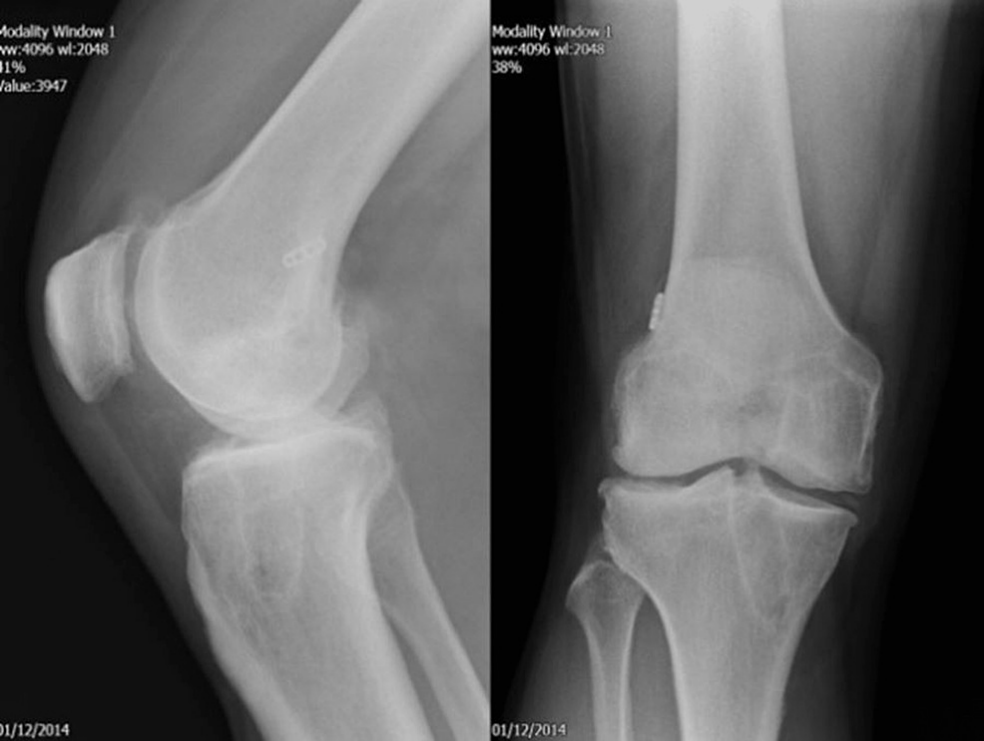
Radiographs of the right knee done in December 2014. Lateral (left) and antero-posterior (right) views showing the tibial and femoral tunnels with a suspensory fixation on the femoral side.

**Figure 2:**
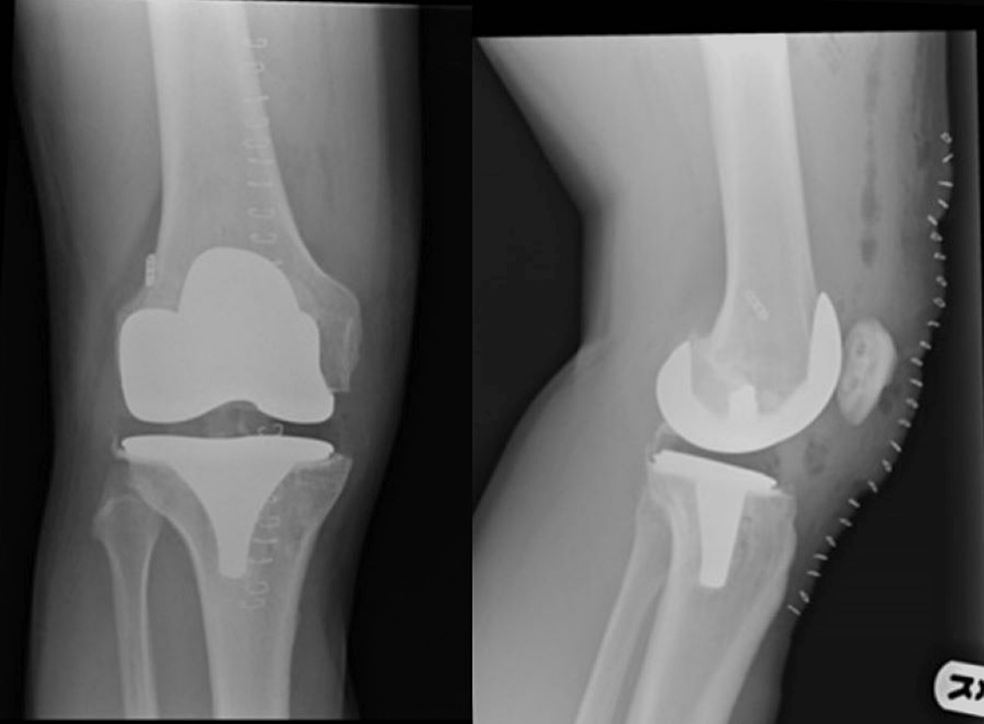
Post-operative radiographs done in February 2016. Antero-posterior (left) and lateral (right) radiographs following primary knee arthroplasty.

A 54-year-old male underwent TKA for arthritis after previous ACLR. ACLR was done 11 years ago, using suspensory fixation and a bio-absorbable screw on the femoral and tibial sides, respectively (Figure 1). Radiographs showed osteoarthritis without any evidence of osteolysis (Figure 1). A cemented TKA (Vanguard, Zimmer Biomet, Warsaw, IN, USA) was undertaken, as shown in Figure 2. During surgery, no osteolytic defects were noted, except the presence of the aperture in the tibial tunnel with the graft remnant. Post-operative recovery was uneventful.

**Figure 3:**
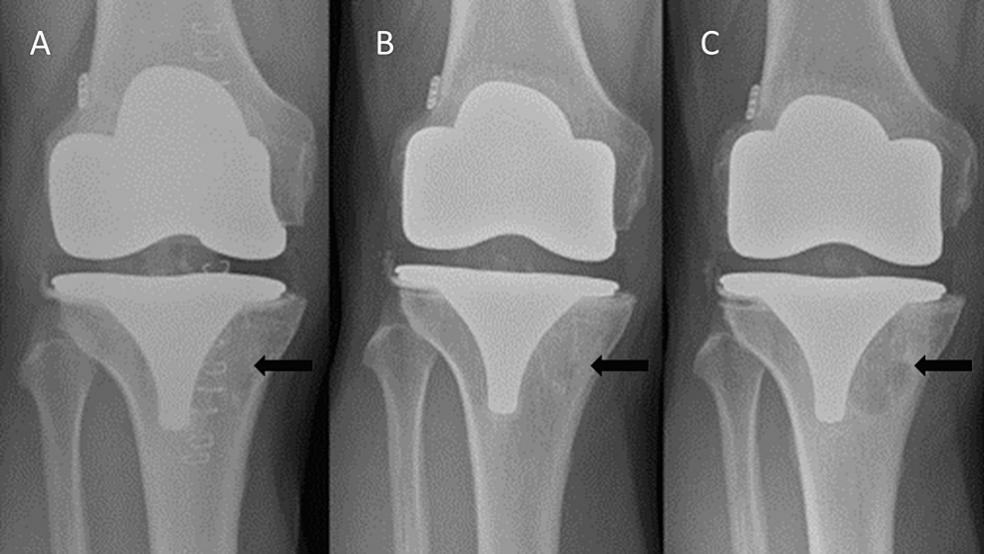
Series of radiographs depicting progressive osteolysis. Osteolysis in comparison depicted by an arrow on antero-posterior radiographs taken in (A) February 2016, (B) July 2017 and (C) June 2019.

**Figure 4:**
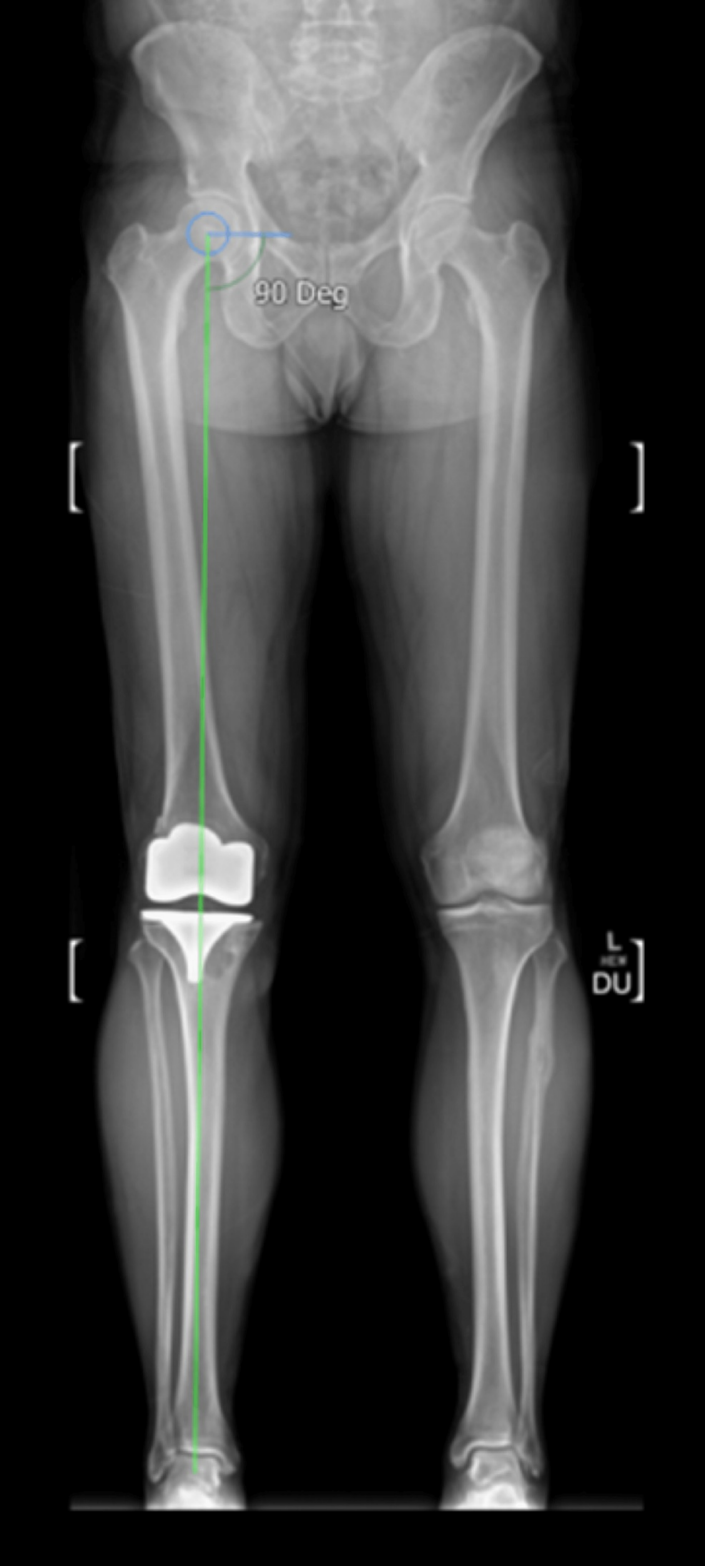
Long leg alignment radiograph before planning revision total knee replacement. The mechanical axis is depicted by the green line drawn from the centre of the femoral head to the centre of the ankle joint.

**Figure 5:**
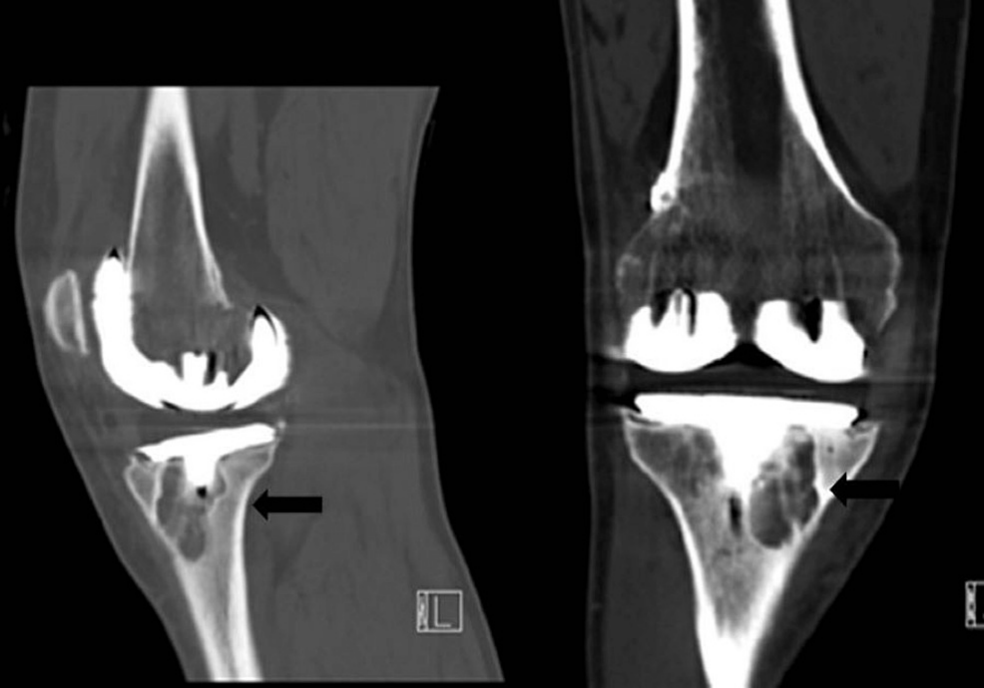
CT scan images. Depicting osteolysis around the tibial component in nine coronal and sagittal views indicated by arrows around the tibial stem. CT, computed tomography

**Figure 6:**
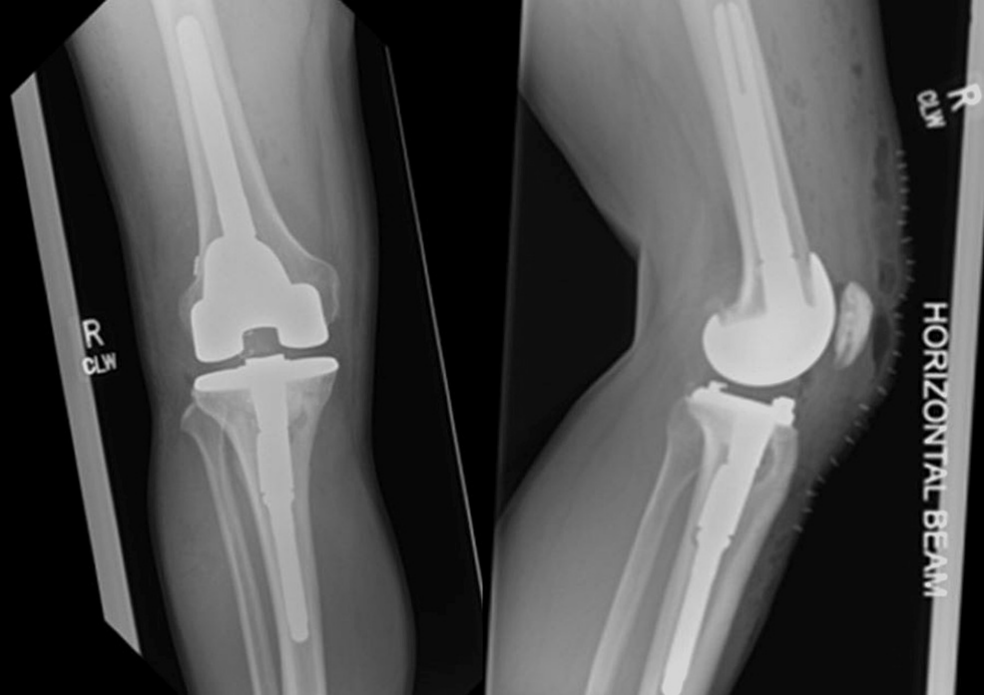
Post-operative radiographs following revision total knee arthroplasty. Antero-posterior (left) and lateral (right) views done in November 2019.

A year after surgery, the patient complained of pain and recurrent swelling. Serial radiographs confirmed progressive osteolysis in the region of tibial tunnel, as shown in Figure 3. An alignment radiograph confirmed restoration of the mechanical axis (Figure 4), whereas CT scans showed a large radiolucency around the tibial component, suggestive of loosening (Figure 5). Serum inflammatory markers were normal, and fluid and tissue sampling of the knee ruled out infection. Post-diagnosis of aseptic loosening, single-stage revision TKR with Vanguard 360 done (Figure 6).

**Figure 7:**
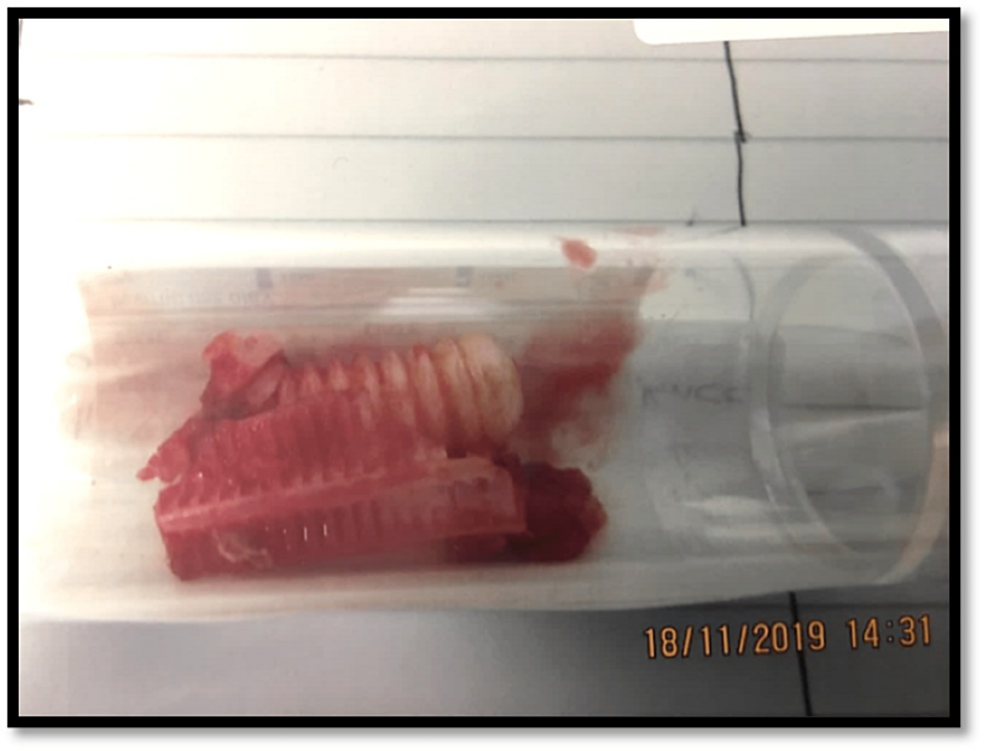
Intraoperative finding showing a fragmented bio-composite screw. The bio-absorbable screw was still present even after years of implantation.

Intraoperatively, after explantation of the loose tibial component, a large cavity filled with jelly-like mass with a fragmented but unabsorbed bio-absorbable screw was encountered (Figure 7). Fluid and tissue samples taken ruled out infection, and histological analysis of the tissue showed mild chronic inflammatory infiltrate predominantly composed of pigment-laden macrophages and occasional giant cells. There was no evidence of a significant polymorph infiltrate. These histological findings were suggestive of a reaction to wear debris and metallosis.

Twenty-two months after surgery, the patient continues to be pain-free with good range of motion.

## Discussion

There have been reports on the higher risk of reoperation after TKA in patients with prior ACLR [2,6,7]. Prosthetic joint infection was reported in two studies [6,7]. Hoxie et al. reported two revisions in TKA patients with prior ACLR, one for instability and one for osteolysis [8]. Massive osteolysis secondary to galvanic corrosion in TKA with previous ACLR has been reported recently [9]. Massive osteolysis is usually associated with significant polyethylene wear and metal-on-metal articulation of the TKA [10].

Bio-absorbable screws introduced into clinical practice in the 1980s were immediately followed by recognition of an adverse tissue response that has the characteristics of an inflammatory foreign body reaction [11,12]. The disadvantages of bio-absorbable screws are low mechanical strength compared to their metallic counterparts, high cost and the development of undesired biologic response [13]. Other complications are intraoperative damage, post-operative late damage and migration into the joint [13].

The absorption of bio-absorbable screws occurs in five stages, during which the screw fragments and these particles are phagocytosed by macrophages releasing by-products [13,14]. The biological response to the degrading bio-absorbable screw is thought to be a result of either a build-up of the acidic degradation products or due to the presence of foreign material [12,13,15]. The material composition of the bio-absorbable screw possibly influences the rate of degradation and absorption and possibly intra-osseous cyst development [15-17]. The absorption rate of poly-L-lactic acid (PLLA) screws is reported to be slower than polyglycolic acid (PGA), which seems to cause a reduced inflammatory response and thus a reduced risk of intra-osseous cyst formation [13].

Bio-absorbable screws, whether composed of a polymer alone or polymer ceramic composites, can cause symptomatic absorption-related intra-osseous tibial tunnel cysts even decades after initial implantation [3]. The delay in the formation of intra-osseous tibial tunnel cysts is usually between 2 and 3 years; however, the development of a cyst 19 years after ACLR has been reported [3]. This unpredictable screw degradation and the reaction to it can result in serious clinical outcomes [13,17].

## Conclusions

Although the exact aetiology is unclear, we hypothesise that the massive osteolysis was caused by the fragmentation of the unabsorbed bio-absorbable screw during the implantation of TKA. This fragmentation evoked an accelerated inflammatory response, leading to osteolysis. The other explanation could be an accelerated inflammatory response due to changes in the surrounding environment of the bio-absorbable screw after TKA.

We advocate caution in TKA patients with prior ACLR and recommend further imaging to rule out tunnel osteolysis. If present, the pathology should be managed by a stemmed tibial component to bypass the defect, which should be curetted and filled with either cement or bone graft, depending upon the size.
